# Modelling lung cancer diagnostic pathways using discrete event simulation

**DOI:** 10.1080/17477778.2021.1956866

**Published:** 2021-08-02

**Authors:** Tracey England, Paul Harper, Tom Crosby, Daniel Gartner, Edilson F. Arruda, Kieran Foley, Ian Williamson

**Affiliations:** a School of Mathematics, Cardiff University, Cardiff, UK; b Velindre Cancer Centre, Cardiff, UK; c Alberto Luiz Coimbra Institute Graduate School and Research in Engineering, Federal University of Rio de Janeiro, Rio de Janeiro, Brazil; d Respiratory Medicine, Aneurin Bevan University Health Board, Newport, UK

**Keywords:** Diagnostic pathway, discrete event simulation, cancer, performance indicators

## Abstract

The United Kingdom has one of the poorest lung cancer survival rates in Europe. In this study, to help design and evaluate a single lung cancer pathway (SCP) for Wales, existing diagnostic pathways and processes have been mapped and then modelled with a discrete event simulation. The validated models have been used to provide key performance indicators and to examine different diagnostic testing strategies. Under the current diagnostic pathways, the mean time to treatment was 72 days for surgery patients, 56 days for chemotherapy patients, and 61 days for radiotherapy patients. Our research demonstrated that by ensuring that the patient attends their first outpatient appointment within 7 days and streamlining the diagnostic tests would have the potential to remove approximately 11 days from the current lung cancer pathway resulting in a 21% increase in patients receiving treatment within the Welsh Government set target of 62 days.

## 1. Introduction

In the United Kingdom, lung cancer is the third most common cancer accounting for 13% of all new cancer cases, and the leading cause of cancer mortality according to Cancer Research UK (2016). Delays in diagnosing lung cancer, before treatment can commence, are known to impact on patient outcomes (Cancer Research UK, 2016). Approximately two-thirds of patients diagnosed with lung cancer begin their treatment within the current 62-day target (UK Lung Cancer Coalition, 2019), with many being diagnosed with advanced stage of disease. The U.K. Lung Cancer Coalition (UKLCC) report “25 by 25” (United Kingdom Lung Cancer Coalition, 2016) found that 65% of health specialists dealing with lung cancer believe that early diagnosis is the most important factor in improving survival rates. Delays are thought to be due to, in part, an increase in the number of urgent referrals, as well as patients following more personalised complex pathways with a wider selection of diagnostic tests available (UK Lung Cancer Coalition, 2019).

In Wales, lung cancer survival is poor because of late-stage diagnosis and treatment. Only 14.5% of Welsh lung cancer patients survive for 5 years or more (Public Health Wales, 2021). The Welsh Government has recently set out its approach to improving cancer services and outcomes with the introduction of a single cancer pathway (SCP) (Welsh Government (2018), Wales Cancer Network (2019)). The SCP is the new target within Wales for diagnosing cancer and starting treatment within 62 days from the date of suspicion of their cancer. The new pathway is designed to replace the previous two-tier pathways (for urgent and non-urgent referrals) and aims to reduce waiting times and improve early diagnosis. The National Optimal Lung Cancer Pathway (NOLCP) was adopted in Wales in August 2017 as a way of streamlining the diagnostic pathway and aims to reduce the time from referral to treatment from 62 to 49 days (UK Lung Cancer Coalition, 2019). Underpinning this is the necessity to understand demand and capacity. The overall aim behind this research was to align capacity to best match demand and to ultimately improve lung patient care and outcomes.

One challenge in estimating the demand and capacity of a healthcare system, particularly when there are potentially multiple diagnostic and care pathways, is the variation in the system (Monks et al. (2016), Zhang (2018)). Discrete event simulation (DES) has been an effective tool for demand and capacity planning across a range of clinical and health delivery services, including breast cancer (Brailsford et al., 2012), colorectal cancer (Harper & Jones, 2005), chlamydia (Viana et al., 2014), HIV (Harper & Shahani, 2003), emergency medical services (Vile et al., 2016), out-of-hour services (Tuson et al. 2018) and trauma and orthopaedic services (England et al., 2019). While DES has been used for cancer screening programmes, there is far less literature on its use for use in specifically modelling cancer diagnostic pathways. For example, Ju et al. (2015) use DES for representing a lung cancer pathway, but their paper does not discuss any aspect of implementation. One possible reason for this, and for the lack of other peer-reviewed studies, is the complexity of the pathway and the variation associated with each patient’s journey (Aspland et al., 2021). Another challenge is the need to capture time-related activities. For example, in the lung cancer diagnostic pathway, we want to include accurate representations of the time needed to arrange and report a diagnostic test for each patient. The baseline model outlined in this study has approximately 80 time-related activities, highlighting the complexity of the lung cancer pathway and the need of an approach, which can provide an accurate representation of a patient’s journey along the pathway. Discrete event simulation provides a very useful way to tackle this complex problem.

Our study therefore provides a case study of how DES has been used to represent the diagnostic pathway for lung cancer patients in a major cancer centre in Wales and to evaluate potential reductions of the time until the start of treatment under different “what-if?” scenarios. In particular, this study is concerned with time savings that can be identified within the diagnostic pathway thus reducing a patient’s time until their diagnosis and their treatment begins. The study does not consider the effect of extra resources (personnel or diagnostic tests) on the current system’s capacity; this could be considered in future research using an adapted form of the simulation model described here.

Our study has supported a wider programme of research to design and implement the Welsh single cancer pathway and to identify areas where gains can be made so that a patient’s time to diagnosis and subsequent treatment can be reduced, thus improving patient outcomes. While this study focuses on hospitals in Wales, it is envisaged that the findings can prove insightful for other locations within the U.K. and internationally.

We present the article in accordance with the STRESS reporting checklist for discrete event simulation modelling (Monks et al., 2019). The STRESS reporting checklist is a standardised approach to improving the reporting of discrete event simulation models so that a simulation study can be reproducible to others interested in carrying out similar research. The checklist describes the purpose of the model, the logic behind the model, the data used, the number of iterations and the simulated run time, and the software/code used to implement the model.

## 2. Methodology

This study focuses on the lung cancer diagnostic pathway within Aneurin Bevan University Health Board (ABUHB). ABUHB provides primary, community, hospital, and mental health services to approximately 600,000 people living in Blaenau Gwent, Caerphilly, Monmouthshire, Newport, Torfaen, and South Powys.

ABUHB was chosen to participate in this study as its cancer services are one of the first in Wales to have implemented Tracker 7 software (specialist cancer services data collection software) into their patient administrative systems (PAS), to track patients prospectively through their entire cancer pathway (Wales Cancer Network, 2017). This case study focuses on the simulation model developed for the lung cancer services at the Royal Gwent Hospital and Nevill Hall Hospital. A thorough analysis of the data collected through the Tracker 7 software was conducted. The data contain 2,995 records for patients referred with suspected lung cancer. The data also contain the dates associated with 1,724 diagnostic tests carried out for these patients. Each row represented a detailed patient record including their longitudinal history of diagnostic tests (dates of referral, dates undertaken, reporting times, etc.) between December 2016 and October 2018.

The referral data also included the dates of their first outpatient appointment, Multi-Disciplinary Team (MDT) meetings, and their decision to treat date. The first treatment that the patient received was also included. Knowing the initial treatment plan allows investigation of whether patients with different treatments spend different lengths of time in the system. The treatment paths considered in the model are chemotherapy (SACT), radiotherapy, surgery, chemoradiotherapy, palliative care, active monitoring, and other (the specific description of this category is not included).

Results of a statistical analysis were used to parameterise the simulation model. Stat-Fit for Simul8 and Easy Fit 5.6 Professional used to estimate the statistical distributions associated with the model parameters. In particular, the model uses the inter-arrival rates, and the service times for each clinic appointment and diagnostic test carried out. A full list of the model parameters can be provided on request.

The total number of referrals, broken down by current pathway (Urgent Suspected Cancer (USC) and non-Urgent Suspected Cancers (nUSC)), and status (active/treated/downgraded), is provided in Table 1. The period of referrals covers 2,995 patients that were referred during the period December 2016 through to the end of October 2018. The percentages are also given. “Active” refers to those patients that are still on the pathway; they may or may not have cancer. “Treated” refers to those patients that have been diagnosed and are receiving treatment (e.g., chemotherapy, radiotherapy, surgery, or supportive care). “Downgraded” refers to those patients that were suspected to have cancer but do not.

**Table 1.** Total number of referrals by USC/nUSC and status (December 2016–October 2018).

Status	USC	%	nUSC	%
Active	72	4.5	580	41.4
Treated	258	16.2	404	28.9
Downgraded	1,265	79.3	416	29.7
Total	1,595		1,400	

Table 2 features the number of diagnostic tests carried out in relation to 1,223 patients with suspected lung cancer. The percentage of the total number of tests is also given for each diagnostic test. Other refers to other tests that the patients might receive such as ECHO, gastroscopies, general biopsies, and other ultrasounds.

**Table 2.** Number of diagnostic tests carried out within ABUHB (December 2016–November 2018).

Diagnostic test	Number of tests	% of tests
Bronchoscopy	113	6.6
X-ray	46	2.7
CT	978	56.7
CT-guided biopsy	187	10.8
EBUS	67	3.9
Lung function test	18	1.0
MRI	32	1.9
PET	122	7.1
US-guided biopsy	46	2.7
Other	115	6.7
Total	1,724	

Further analysis of the diagnostic test data on Tracker 7 shows that 98% of patients (1,197 of 1,223) have three or fewer diagnostic tests (Table 3) with the first test most likely to be a CT scan. For patients treated with curative intent, the most likely diagnostic pathway was an initial CT followed by a PET-CT and biopsy.

**Table 3.** Number of tests per patient (December 2016–November 2018).

Number of tests	Number of patients	% of patients
1	852	69.7
2	277	22.7
3	68	5.6
4	19	1.6
5	5	0.4
6	1	0.08
7	1	0.08
Total tests	1,724	
Total patients	1,223	

Considering the diagnostic tests, we define

***Time to arrange***

The time between the request date and the date when the test was carried out.

***Reporting time***

The time between the test taking place and the report being produced.

***Total time***

The sum of the ***Time to arrange*** and ***Reporting time***.

The average times associated with the most prevalent tests used in ABUHB are shown in Table 4. This shows that the largest delays in arranging a test are associated with MRIs and PET-CTs while the longest delays in reporting are related to bronchoscopies and X-rays. The Tracker 7 data for each of the diagnostic tests listed in Table 4 has been used to parameterise the service times in the model.

**Table 4.** Time associated with diagnostic tests (in days), correct to 1 d.p.

Diagnostic Test	Time to arrange	Reporting time
Bronchoscopy	5.9	5.8
CTs^1^	9.9	5.4
CT-guided biopsy	8.5	2.0
EBUS	4.3	1.1
LFT	8.0	-
MRI	11.3	3.7
PET	10.2	1.9
US-guided biopsy	9.4	1.2
X-ray	0.4	5.8

### 2.1. Model

We have built a detailed computer simulation model (using SIMUL8 software) to capture the diagnostic pathway for lung cancer patients within ABUHB. The model allowed us to simulate individual patients on their diagnostic pathway. The sequence of tests in the current lung cancer pathway simulation model is based on the Tracker 7 data alongside input from respiratory consultants and specialist nurses within the health board.

**Figure 1.**
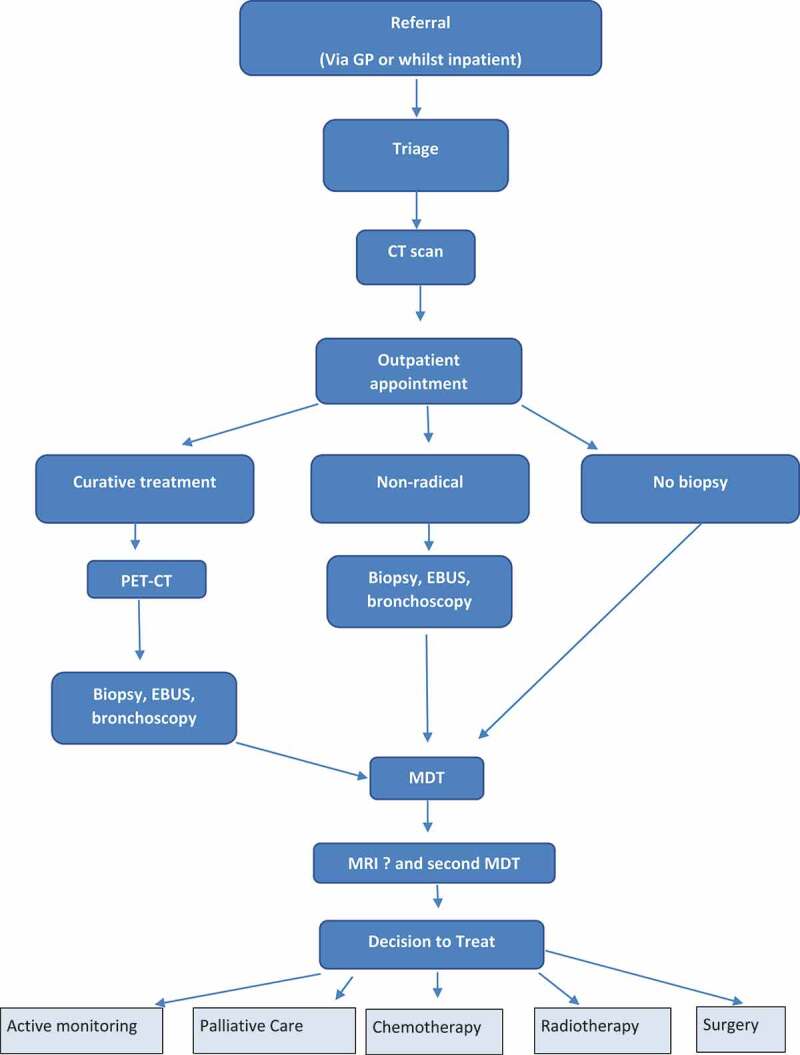
Current Lung Cancer Pathway.

Under the current lung cancer pathway (Figure 1), a patient is referred and then triaged. Patients are then sent for a CT scan and the results are discussed with the patient at an outpatient clinic. Patients suitable for radical curative treatment (e.g., surgery, chemotherapy (SACT) or radiotherapy) are referred for further combinations of diagnostic tests, usually starting with a PET-CT and then a biopsy (CT-guided or US guided), EBUS or bronchoscopy depending on radiological findings. The results are discussed at an MDT where the proposed treatment is recommended. Some patients will require an MRI and further discussion at a second MDT. The patient will then attend a further clinic appointment and their treatment options will be discussed and management plan agreed. Patients that are not suitable for radical curative treatments may also be referred for further tests before their case is discussed and they receive either active monitoring or palliative care.

**Figure 2.**
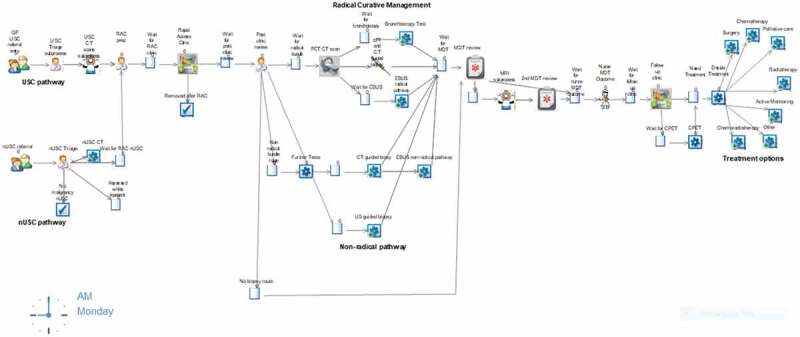
Screenshot of the Current Lung Cancer Pathway Model.

The simulation model for the current lung cancer pathway (Figure 2) considers the patient’s pathway from their point of referral to the time that their treatment starts.

The final section of the simulation model considers the steps involved in each of the treatment options until the patient starts their treatment. For patients undergoing curative treatment, they undergo an outpatient clinic where they consent to their treatment. Following this, the planning and preparatory processes are carried out and these have been captured in the simulation. For example, detailed contour planning is needed for radiotherapy patients to ensure that the correct dose of radiation is applied to the correct area. In the case of chemotherapy patients, blood tests are required to ensure that the white blood count and liver and kidney function are at a sufficient level for the oral or IV drug that will be prescribed. Surgical patients are required to undertake a pre-assessment clinic before they are admitted to hospital for surgery.

The simulation model monitored patient progress along the pathway. The model captured the average time in the system and the percentage of patients that are within a certain time threshold. In the current lung cancer pathway model, the time in the system represents the time between the point of referral (POR) by the general practitioner and the time of the first treatment. The target is 62 days.

As the pathways are different for patients undergoing different treatment options, the results were captured for each of the main treatment categories (SACT, radiotherapy, and surgery) along with palliative care and active monitoring. The numbers of patients classified as either undergoing chemoradiotherapy treatment or other are minimal and not considered in the scenario analysis. In the scenario analysis, the results for the SACT, radiotherapy, and surgery pathways are presented and discussed.

### 2.2. Model evaluation

The simulation model was run for 300 iterations to ensure stable predictions of the key performance indicators (KPIs). The KPIs collected for the current lung cancer pathway were:

1. The average time spent on the lung cancer pathway (with 95% confidence intervals)
2. The percentage of patients that spent 62 days or less on the pathway (with 95% confidence intervals)

Model validation and verification of the current pathway model ensures that the model sufficiently mimics the current lung cancer pathway implemented within ABUHB. The KPIs from the model were validated against the actual data (Table 5). The model results are very close to the observed values. As well as the key performance indicators, the number of referrals, diagnostic tests, MDT meetings, outpatient clinics and the number of patients on each treatment pathway were recorded and compared with the data to validate the model. The model’s accuracy in predicting these quantities was above 95%, suggesting that the model was a sufficiently good representation of the current lung cancer pathway within ABUHB.

**Table 5.** Model validation: comparing the KPIs from the model with the observed values (correct to 1 d.p.).

KPI	Observed	Model
SACT (Mean time in system)	56.5	55.3 (55.0–55.6)
Radiotherapy (Mean time in system)	61.5	62.6 (62.0–63.2)
Surgery (Mean time in system)	71.7	67.3 (66.6–67.9)
% of SACT patients within 62 days	67.2	67.1 (66.5–67.7)
% of Radiotherapy patients within 62 days	56.6	54.9 (53.9–55.9)
% of Surgery patients within 62 days	47.6	47.5 (46.5–48.4)

### 2.3. Scenarios

Scenario analyses were also conducted to aid future demand and capacity decisions. For example, scenarios considered the benefits associated with different levels of service provision for the diagnostic tests. Currently, in Wales, there are lengthy delays associated with certain diagnostic tests which can add 2–3 weeks to the patient’s pathway (see Table 4). This of course is not ideal given the aim to achieve a 62-day target for the start of treatment from the point of suspicion for 95% of the patients. Our scenarios were selected after careful consideration with collaborating clinicians and nurses. The full list of the 11 scenarios and their descriptions is given in Table 6.

**Table 6.** Scenarios and their descriptions.

Scenario number	Scenario	Description	Difference to the baseline model
1	Outpatients within 7 days	All patients seen in an outpatient clinic within 7 days of their date of suspicion, having had their CT scan.	Shorter time to first outpatient appointment
2	7 days between tests	A weekly diagnostic test	Reduced time to arrange test
3	3 days between tests	A bi-weekly test	Shorter time to test
4	1 day between tests	A daily test service	Next day test
5	Tests reported within 2 days	A diagnostic test is reported within 2 days of it being conducted.	Reduced reporting time
6	Tests reported within 1 day	The results of the test are reported the next day.	Next day results
7	Outpatients within 7 days, 3 days between tests and reported within 2 days:	The patient is seen within 7 days of their date of suspicion and that tests are offered twice a week with the reports available within 2 days of the test.	Shorter time to clinic, quicker diagnostic test and results
8	Outpatients within 7 days, 1 day between tests and next day report	The patient is seen within 7 days and that tests are offered daily with results available the next day	Shorter time to clinic, next day testing and results
9	Outpatients within 7 days, 3 days between tests, reported within 2 days, next day chemo outpatient clinic after MDT and next day bloods	Scenario 7 with next day chemotherapy outpatient clinic after the MDT and next day blood tests.	Shorter time to clinic, quicker diagnostic test and results with improved chemotherapy pathway
10	Outpatients within 7 days, 3 days between tests, reported within 2 days, improved radio pathway after MDT	Scenario 7 with improved radiotherapy pathway after MDT	Shorter time to clinic, quicker diagnostic test and results with improved radiotherapy pathway
11	Outpatients within 7 days, 3 days between tests, reported within 2 days, improved surgery pathway after MDT	Scenario 7 with improved surgery pathway after MDT	Shorter time to clinic, quicker diagnostic test and results with improved surgery pathway

In the first scenario (**Scenario 1: *Outpatients within 7 days***), we considered the effect of all patients being seen in an outpatient clinic within 7 days of their date of suspicion, having had their initial CT scan.

In the next three scenarios (**Scenarios 2, 3, and 4**), we considered the effect of different levels of service provision in the diagnostic tests. In the ***7 days between tests*** scenario we considered the effect of a weekly diagnostic service. In the *3 days **between tests***, we considered a bi-weekly service and in the ***1 day between tests***, a daily service.

The next two scenarios, Scenarios 5 and 6 (***tests reported within 2 days, tests reported within 1 day***) consider the effect of reducing the time it takes to report a diagnostic test to 2 days or less. Table 4 shows that the current time to produce a report can be up to 5 days for certain diagnostic tests.

The next two scenarios, Scenarios 7 and 8, show a combination of strategies to reduce the time until treatment:

***Scenario 7***

***Outpatients within 7 days, 3 days between tests and reported within 2 days***: ensuring the patient is seen within 7 days of their date of suspicion and that tests are offered twice a week with the reports available within 2 days of the test.

***Scenario 8***

***Outpatients within 7 days, 1 day between tests and next day report***: ensuring the patient is seen within 7 days and that tests are offered daily with results available the next day.

Further scenarios were considered that examined the effect of reducing the time after a patient’s case was discussed at an MDT and before they started their treatment. The aim of the National Optimal Lung Cancer Pathway is that patients should begin their treatment within 21 days of their decision to treat. In each of the three treatment paths, scenarios were considered where the patient was seen in an outpatient clinic 1 day after their case was discussed in the MDT. With chemotherapy (SACT) patients, Scenario 9 considered the next day outpatient clinic and a more streamlined blood test service. For radiotherapy patients, Scenario 10 considered the next day outpatient appointment coupled with a reduction in the time taken to send out the appointment letters to the patient. For surgery patients, Scenario 11 considered a reduced wait for both their pre-assessment clinic and admission to the hospital.

Considering all of these scenarios could examine whether all patients could potentially receive treatment within the 62-day target proposed by the Single Cancer Pathway and possibly the future 49-day target.

## 3. Results

The results for the baseline and scenario models for the current lung cancer pathway are presented. Table 7 shows the mean time to treatment on the current lung cancer pathway under the baseline and scenario models. The results have been separated according to the treatment that the patient is designated to receive: SACT, radiotherapy or surgery. The observed mean time to treatment (from the data) is shown in the “Data” column. The estimated mean time to treatment (from the model) is given as well as the 95% confidence intervals following the 300 iterations of each model run.

**Table 7.** Comparing the average time on the current pathway (in days), correct to 1 d.p.

	Data	Baseline	Outpatients within 7 days	7 days between tests	3 days between tests	1 day between tests	Tests reported within 2 days	Tests reported within 1 day	Outpatients within 7 days, 3 days between tests and reported within 2 days	Outpatients within 7 days, 1 day between tests and next-day report^2^	Outpatients within 7 days, 3 days between tests, reported within 2 days, next day chemo outpatient clinic after MDT and next day bloods	Outpatients within 7 days, 3 days between tests, reported within 2 days, improved radio pathway after MDT^3^	Outpatients within 7 days, 3 days between tests, reported within 2 days, improved surgery pathway after MDT^4^
SACT	56.4	55.3 (55.0–55.6)	50.1 (49.8–50.3)	52.6 (52.4–52.9)	48.4 (48.2–48.6)	46.0 (45.8–46.2)	53.9 (53.6–54.2)	53.0 (52.7–53.2)	46.2 (46.0–46.4)	44.3 (44.1–44.6)	31.1 (31.0–31.3)	46.2 (46.0–46.4)	46.2 (46.0–46.4)
Radiotherapy	61.5	62.6 (62.0–63.2)	57.5 (56.9–58.1)	60.0 (59.4–60.6)	55.8 (55.2–56.4)	53.3 (52.7–53.9)	61.2 (60.6–61.8)	60.2 (59.6–60.8)	53.6 (53.0–54.2)	51.7 (51.1–52.3)	53.6 (53.0–54.2)	39.1 (38.9–39.2)	53.6 (53.0–54.2)
Surgery	71.7	67.3 (66.6–67.9)	62.3 (61.6–62.9)	64.7 (64.1–65.4)	60.5 (59.9–61.1)	58.1 (57.5–58.7)	65.9 (65.3–66.5)	64.9 (64.3–65.6)	58.4 (57.8–59.0)	56.5 (55.9–57.1)	58.4 (57.8–59.0)	58.4 (57.8–59.0)	35.4 (35.3–35.6)

The first scenario (***Outpatients within 7 days***) considered the effect of ensuring that patients are seen in an outpatient clinic within 7 days of their date of suspicion. If this could be achieved, then 5 days could be removed from the lung pathway. For example, the mean time to diagnosis for surgery patients would reduce from 67 to 62 days (see Table 7).

The second scenario, ***7 days between tests***, sees that providing a weekly diagnostic service provides a minor improvement with approximately 3 days removed from the current pathway. However, a ten-day reduction can be achieved if a daily diagnostic service (***1 day between tests*** scenario) is available.

Reducing the reporting times associated with diagnostic tests to 2 days (***Reported within 2 days***) sees a one to two-day reduction in the pathway length. If the reports were available the day after the test (***Reported within 1 day***), patients could see two days removed from their pathway.

The combined scenarios offer the largest reduction in the pathway length with approximately 9 days removed from the pathway if a patient is seen within 7 days and they are offered diagnostic tests (provided twice a week) and receive the results within 2 days. Eleven days can be removed from the current lung cancer pathway if patients are seen in clinic within 7 days and a daily diagnostic service on offer with reports available the next day.

If we combine these scenarios with a time to treatment that is reduced by a quicker outpatient appointment and further tests, we can achieve compliance with the National Optimal Lung Cancer Pathway; 95% of patients diagnosed and start treatment within 62 days. If patients are seen in clinic within 7 days, have tests that are offered twice a week and reported within 2 days, and start their treatment within 21 days, the scenario suggests that the average time in the system drops to 40 days or under for all treatment pathways in each hospital.

Table 8 summarises the effect of each scenario on the mean time in the system when compared with the baseline model. The most dramatic reductions occur when a combination of interventions are used. For example, seeing the patient within 7 days of the date of suspicion, offering bi-weekly diagnostic tests where the results are received within a couple of days and improving the initial stages of the treatment pathway following the MDT where the patient’s diagnosis is discussed.

**Table 8.** The effect of the scenario on the mean time in the system.

Scenario number	Scenario	Effect (number of days removed)
1	Outpatients within 7 days	5
2	7 days between tests	3
3	3 days between tests	7
4	1 day between tests	10
5	Tests reported within 2 days	1– 2
6	Tests reported within 1 day	2
7	Outpatients within 7 days, 3 days between tests and reported within 2 days:	9
8	Outpatients within 7 days, 1 day between tests and next day report	11
9	Outpatients within 7 days, 3 days between tests, reported within 2 days, next day chemo outpatient clinic after MDT and next day bloods	25
10	Outpatients within 7 days, 3 days between tests, reported within 2 days, improved radio pathway after MDT	22
11	Outpatients within 7 days, 3 days between tests, reported within 2 days, improved surgery pathway after MDT	36

Table 9 shows the baseline and scenario results for the percentage of patients that spend 62 days or less on their pathway to treatment under the current system. The percentage is given along with the 95% confidence intervals following the 300 iterations of a model run for a given baseline or scenario experiment. The current target is 95%. Under the “Data” column, which shows the observed percentages calculated from the data, we observe that under the current lung cancer pathway, none of the treatment pathways achieve this target. The SACT group performs the best with almost two-thirds of patients achieving the 62-day target.

**Table 9.** Percentage of pathways up to 62 day long (current pathway), correct to 1 d.p.

	Data	Baseline	Outpatients within 7 days	7 days between tests	3 days between tests	1 day between tests	Tests reported within 2 days	Tests reported within 1 day	Outpatients within 7 days, 3 days between tests and reported within 2 days	Outpatients within 7 days, 1 day between tests and next-day report	Outpatients within 7 days, 3 days between tests, reported within 2 days, next day chemo outpatient clinic after MDT and next day bloods	Outpatients within 7 days, 3 days between tests, reported within 2 days, improved radio pathway after MDT^5^	Outpatients within 7 days, 3 days between tests, reported within 2 days, improved surgery pathway after MDT^6^
SACT	67.2	67.1 (66.5–67.7)	76.9 (76.4–77.4)	71.8 (71.2–72.4)	81.3 (80.8–81.8)	85.9 (85.3–86.4)	69.7 (69.2–70.3)	71.4 (70.9–72.0)	85.5 (85.1–86.0)	89.0 (88.6–89.4)	99.0 (98.9–99.1)	85.5 (85.1–86.0)	85.5 (85.1–86.0)
Radiotherapy	56.6	54.9 (53.9–55.9)	64.5 (63.4–65.7)	58.4 (57.3–59.5)	67.6 (66.4–68.8)	72.9 (71.7–74.1)	57.6 (56.5–58.6)	59.3 (58.3–60.4)	72.0 (70.8–73.2)	76.1 (74.9–77.3)	72.0 (70.8–73.2)	95.8 (95.6–96.1)	72.0 (70.8–73.2)
Surgery	47.6	47.5 (46.5–48.4)	55.6 (54.6–56.7)	49.8 (48.8–50.9)	57.6 (56.5–58.7)	62.7 (61.5–63.8)	49.4 (48.5–50.4)	50.9 (49.9–51.9)	62.0 (60.8–63.1)	65.8 (64.6–67.0)	62.0 (60.8–63.1)	62.0 (60.8–63.1)	98.2 (98.0–98.3)

Under the first scenario (***Outpatients within 7 days***) where patients attend an outpatient clinic within 7 days, the percentage of patients receiving their first treatment would increase by 8–10% (see Table 9). For example, under the current system, approximately 47.49% of surgery patients receive their first treatment within 62 days. However, this increases to 55.61% if patients can be seen in the outpatient clinic within 7 days of their point of suspicion.

Under the second scenario (***7 days between tests***) where a weekly diagnostic service is offered, there is a slight increase in the percentage of patients that receive their treatment within 62 days at both hospitals. Patients would observe a 2–4% increase. If a daily diagnostic service (***1 day between tests*** scenario) is offered, there is a larger percentage of patients receiving their treatment within 62 days; an increase of between 15% and 18%.

Under the improved reporting scenarios (***Reported within 2 days; Reported within 1 day***), approximately 2–4% more patients would receive treatment within 62 days of their date of suspicion.

If patients could be seen in an outpatient clinic within 7 days and then be offered a daily diagnostic slot with the report available the next day, the service would see an 18–20% increase in the number of patients starting treatment within 62 days of their date of suspicion.

Combining the bi-weekly testing scenario with a more streamlined time to treatment could see almost all patients diagnosed and start their treatment within 62 days. If patients could be seen within 7 days of their date of suspicion, have a bi-weekly test and an improved treatment path after their MDT then the improved 49-day target suggested by the Single Cancer Pathway could be achieved in all of the main treatment routes. This emphasises the need to address all parts of the current pathway and improve it where possible.

## 4. Discussion

This case study has used discrete event simulation to represent the current lung cancer pathway from point of referral through to the start of treatment. The model provided an accurate representation of the system and resources currently used. Scenario analysis showed that streamlining the diagnostic tests with same- or next-day reporting and reducing the time until the initial outpatient clinic each have significant benefits in reducing the time a patient spends on the current lung cancer pathway. However, combining the scenarios sees a much-reduced time on the diagnostic pathway, especially when the time after the MDT meeting and prior to the start of treatment is reduced to 21 days or less.

Discrete event simulation modelling proved useful in providing a means of representing a complex pathway in a virtual environment, which can be analysed through “what-if?” scenarios. Detailed statistical analysis of the Tracker 7 lung cancer data alongside expert opinion has been used to ensure the model was an accurate a representation of the current system.

Typically, in developing simulation models, an important part is to build and validate the model with key personnel that work in the appropriate service. A major strength of this study was that information from respiratory consultants and specialist cancer nurses was used alongside the data to produce the process map that formed the structure of the simulation model. Another benefit of the simulation is that the visual representation of the current model by a series of images for each activity ensures that the process and results can be understood by clinicians, administrators, policy makers and analysts, thus making it a useful decision support tool.

In the scenario analysis, the following areas of improvement were identified:

1. Reducing the time until the patient is first seen in clinic to under 7 days
2. Offering daily diagnostic tests
3. Reporting the results within a day of the diagnostic test
4. Reducing the time until the first treatment by offering a next-day outpatient clinic where the proposed treatment is discussed coupled with shorter times to planning and blood tests.

To facilitate a more streamlined diagnostic service:

- ABUHB should continue to reserve a couple of CT scan appointments, each day, for new lung cancer patient referrals and ensure that results are ready for the first outpatient clinic appointment (which should be within 7 days of the date of suspicion).
- Rapid access to subsequent investigations, with rapid reporting turnaround times, with tests done in parallel rather than sequentially.
- Radiologists would need to be available to provide same-day/next-day reports on the scans.
- There would be a need to increase the number of radiographers and radiologists providing the diagnostic tests so that the turnaround times of each test are reduced. Adding resources to the current simulation model could be considered in future research.
- The time between the MDT to DTT should be a few days at most. In the National Optimal Lung Cancer Pathway, it is at most 3 days.
- There would also be a need to ensure that the time between the final MDT and the treatment starting is kept to a minimum with waiting lists being managed appropriately so that the waiting time does not exceed 21 days. The single cancer pathway stipulates that patients should start treatment within 21 days of their decision to treat.

Further examination of the radiographers and radiologists’ workload is also needed to see how daily tests and same-day/next-day reporting can be achieved in the future. Current research is considering how the capacity of diagnostics would need to change to support the single cancer pathway in Wales. Our findings have been presented back to ABUHB cancer services and the programme board for the Cancer Research UK grant that funded this work and the wider recommendations are being reported to Welsh Government for further consideration.

## Notes

1. Including abdomen, thorax, head, colon, TAP, urinary tract.

2. Patients are seen in outpatients with CT results within 7 days and further tests are 1 day apart and the time to report is 1 day.

3. Next day outpatient appointment after MDT, reduced time to produce contour.

4. Next day outpatient appointment after MDT, reduced time to PAC clinic and admission.

5. Next day outpatient appointment after MDT, reduced time to produce contour.

6. Next day outpatient appointment after MDT, reduced time to PAC clinic and admission.
